# Psychometric Properties of The Greek Version of the Test of Performance Strategies-Competition Scale (TOPS-CS)

**DOI:** 10.2478/v10078-011-0047-4

**Published:** 2011-10-04

**Authors:** Christos Katsikas, Olyvia Donti, Maria Psychountaki

**Affiliations:** 1 Department of Physical Education and Sport Sciences, University of Athens, Greece

**Keywords:** psychological skills, athletes, validity, reliability, competition

## Abstract

The aim of the present study was to examine the psychometric properties of the Test of Performance Strategies-Competition scale (TOPS-CS; Thomas et al., 1999) in Greek athletic population. The TOPS-CS was designed to assess eight psychological strategies used by athletes in competition (activation, automaticity, emotional control, goal-setting, imagery, negative thinking, relaxation and self-talk). In order to evaluate the psychometric properties of the inventory, two different research studies were conducted in two different age groups (n_1_=382 athletes, aged 16 to 20 years and n_2_=343 athletes, aged 12 to 15 years). Furthermore, 263 athletes, (aged 16 to 20 years) completed the TOPS-CS, purposing to perform confirmatory factor analysis. The results of the first study supported the initial factorial structure of the TOPS–CS for athletes aged 16–20 years. Reliability analysis also provided adequate evidence for the internal consistency and stability of the scale for Greek athletes of this age. However, for athletes aged 12 to 15 years, the validity and reliability of the inventory were questionable and further research is required.

## Introduction

It is a common belief among sport specialists that successful athletic performance is strongly influenced by the psychological skills of the athletes (Gould et al., 2002; Hardy et al., 1996). In addition, it is reported that the use of psychological skills during the first stages of athletic development may promote more efficient athletic skills once the athlete matures (Lane et al., 2004; Williams & Reilly, 2000). Hence, researchers have pointed out the benefits of incorporating psychological skills’ training into youth sport programmes (Hanton & Jones, 1999; Harwood & Swain, 2001).

The assessment of the athlete’s psychological skills is an integral part of the work of sport psychologists (Balague, 1999; Taylor, 1995; Thomas et al., 1999). For sport psychologists, it is important to examine and understand what psychological processes might be contributing to quality of performance, to explore the development and use of psychological skills during all the stages and levels of athletes’ career and to examine the link between the practice of psychological skills in training and in competition.

One way of measuring athlete’s psychological skills is to use questionnaires or inventories. Over the last years, several psychological skills inventories have been proposed, as the ‘Psychological Skills Inventory for Sports’ (PSIS; Mahoney et al., 1987) and the ‘Athletic Coping Skills Inventory - 28’ (ACSI-28; Smith et al., 1995). The utility of such instruments depends fundamentally upon their psychometric properties. If validity and reliability have not been clearly demonstrated, results coming out from the use of these tools are doubtful (Lane et al., 2004). Thomas et al. (1999) pointed out that the validity of some of the most popular existing inventories have not been demonstrated beyond doubt and developed a new inventory, the ‘Test of Performance Strategies’ (TOPS). According to its creators, TOPS was “based upon the psychological processes thought to underlie successful athletic performance” (Thomas et al., 1999). It was assumed that it is important to distinguish strategies used in competition from those used during practice, as they are two different contexts of the athlete’s life.

TOPS-Competition Scale was designed to assess the psychological skills and strategies used by athletes in competition. It consists of eight factors, i.e. self-talk, emotional control, automaticity, goal-setting, imagery, activation, relaxation and negative thinking.

Several researchers (Jackson et al., 2001; Neil et al., 2006; Gould et al., 2002) have recommended the use of the Test of Performance Strategies (TOPS; Thomas et al., 1999) as an effective instrument for assessing adult athletes’ psychological skills. This inventory has also been used to examine the relationships between psychological skills and issues such as top performance (Taylor et al., 2008), flow (Jackson et al., 2000; Jackson et al., 2001), anxiety (Neil et al., 2006; Hayslip et al., 2010), mental toughness (Jones et al., 2002), and emotions (Cohen et al., 2006).

Despite the fact that TOPS has shown satisfactory factorial validity, Thomas et al. (1999) pointed out the need to test the measurement model using confirmatory techniques. Lane et al. (2004) examined the factorial validity of the instrument, using confirmatory factor analysis, with data from adolescent athletes. Results provided partial support for the overall measurement model for the competition items. The subscales of automaticity, goal-setting, relaxation and self-talk showed good fit whereas activation, emotional control, imagery and negative thinking did not.

Thomas et al. (1999), developing the inventory, used a large sample of different age range athletes, representing many sports and competing in a variety of performance levels. The heterogeneity of the initial sample showed its possible applicability across a broad range of age and performance levels (Lane et al., 2004). However, the possibility of generalisation in athletes of different age groups is limited. According to Schutz and Gessaroli (1993), researchers should test the generalizability of measures for different populations of interest. In addition, few studies have evaluated the validity of TOPS for adolescent and young adolescent athletes (Lane et al., 2004) though in some sports (gymnastics, figure skating, diving, and swimming) the competing period between 12 to 15 years is considered to be critical for athletes’ future evolution (Arkaev & Sutsilin, 2004).

Since psychological skills contribute to a successful performance, coaches and sport psychologists could benefit from a valid and reliable instrument of assessing athletes’ mental skills. Possible problems in the use of psychological skills, such as difficulties in maintaining concentration, regulating arousal levels or setting goals could be recognized and improved in the early stages. The purpose of this study was to check the psychometric properties of the TOPS-Competition scale in Greek athletic population. The validity and reliability of the instrument were examined with exploratory factor analysis in two different athletes’ age groups (aged 16–20 and 12–15 years old) and confirmatory techniques were also used to check the measurement model.

## Methods

### Participants

Three groups of athletes participated in the study in order to evaluate the psychometric properties of the TOPS-CS, in Greek athletic population. Athletes were competing in a broad variety of individual and team sports and had different levels of competing experience.

### Exploratory factor analysis

#### Group 1:

National and international level athletes (n_1_=382) (222 males, 160 females), aged 16–20 years (M=19.97±SD=2.40 years), training in sport clubs and national teams affiliated with the Greek Sports Federations, from 25 different sports.

#### Group 2:

National and international level athletes (n_2_=343) (256 males, 87 females), aged 12–15 years (M=13.68, ±1.39 years), training in sport clubs and national teams affiliated with the Greek Sports Federations, from 14 different sports.

### Confirmatory factor analysis

#### Group 3:

National and international level athletes (n_3_=288) participated in the third study (178 males and 110 females), aged 16–20 years (M=19.57 ±1.98 years) from 25 different sport disciplines.

As a condition of participation in the research, all athletes had to have at least two years of competitive experience.

### Measurement instrument

The Test of Performance Strategies-Competition scale (TOPS-CS; Thomas et al., 1999) is a 64-item self-report instrument designed to assess the psychological skills and strategies used by athletes in competition and during practice. It consists of two scales, competition and practice and each one of them of eight subscales. Seven factors are common to both competition and practice scales; self-talk (maintaining a positive internal dialogue), emotional control (controlling emotions under pressure), automaticity (performing with little conscious effort), goal-setting (setting personal goals), imagery (visualizing sport performance), activation (maintaining an optimal level of arousal), relaxation (practicing to remain calm under pressure). The eighth factor, negative thinking (thoughts of failure), replaces attentional control (remain concentrated on the task) in the competition scale, as a competition specific factor. Each subscale consists of four items. Answers are given on a 5-point Likert scale anchored by 1 (never) to 5 (always).

### Translation

The translation of the TOPS into Greek included the following procedure: first, 5 interpreters specialized in the issues of sport psychology conducted a back and forth translation, that involved the translation of the TOPS into Greek and then into the original version. Second, two pilot studies were contacted (20 athletes, aged 16 to 20 years and 20 athletes, aged 12 to 15 years) to examine problems in the content validity of the inventory.

### Administration of the Test

Adult athletes provided informed consent and volunteered to take part in this study. For adolescent athletes, a written parental consent for participation in the study was provided. Instructions for the participants included a reminder to respond to all items and a statement that there were no right or wrong answers.

The participants filled in the inventory before or after their training sessions.

## Results

### Exploratory Factor Analyses

The value 0.833 of Kaiser-Meyer-Olkin measure of sampling adequacy confirmed that the choice of exploratory factor analysis was justified. A principal component factor analysis (Varimax rotation with Kaiser Normalisation) was performed to determine the number of factors. Only factors with an eigenvalue of at least one were extracted.

Results supported the initial factorial structure of the TOPS-CS for athletes aged 16 to 20 years. Principal components analysis produced eight factors with loadings from .45 to .90, communalities .40 to .84 and total variance explained of 63.27% (Table 1). It is noteworthy to mention that factors were produced with free selection of factors and that all factors contained all the items of the initial scale.

For athletes aged 12 to 15 years, results did not support the initial factorial structure of the TOPS-CS. Principal component analysis produced 7 factors as the items of the goal-setting factor were loading in other factors as well, mostly in the factor of activation. Furthermore, the automaticity factor demonstrated inadequate content validity.

### Internal Consistency and Reliability

Reliability analysis of the first study provided adequate evidence for the internal consistency and stability of the scale for Greek athletes aged 16–20 years. Cronbach’s *α* values for all factors ranged from .63 to .84. Test-retest reliability with an interval of 2 to 4 weeks (n=120) ranged from .64 to .81 (Table 2).

However, for athletes aged 12–15 years reliability indices were not acceptable. Cronbach’s *α* values for the seven produced factors ranged from .42 to .51 and test-retest reliability values from .41 to .51.

### Confirmatory factor analysis

Confirmatory factor analysis, using a different sample (n_3_=288) of athletes, was conducted to confirm the previously obtained factorial structure. The confirmatory factor analysis was conducted with a computer program Analysis of Moment Structures (AMOS; Arbuckle, 1997).

The primary index used for model fit was the “root mean square error of approximation” (RMSEA), which is a measure of the mean discrepancy between the observed covariances and those implied by the model per degree of freedom. Values less than 0.05 are indicators of a good fit. Certain researchers consider 0.08 as an acceptable cut-off value, but certainly an RMSEA value above 0.1 indicates a poor model fit. Two additional incremental fit indices are reported: TLI and CFI. The TLI, (Tucker-Lewis coefficient), belongs to the family of indices that compare the discrepancy of the specified model in comparison to the baseline model (Bentler & Bonett, 1980; Bollen, 1989). The typical range for TLI lies between 0 and 1, but it is not limited to that range. TLI values close to 1 indicate a very good fit. A value of TLI=0.9 is considered a cut-off value, above which there is an indication of a good model fit. The same criteria apply for the CFI (comparative fit index). The confirmatory factor analysis for the overall model gave an RMSEA value of 0.049, with TLI=0.892 and CFI=0.911, providing acceptance for the structure of the inventory. Following the analysis for the total model, separate confirmatory factor analyses were performed for each factor (Table 3). Table 3 shows the fit indices of confirmatory factor analysis for the model fit of each individual factor. The RMSEA values for the factors activation, automaticity, and self talk are above the value of 0.1.

## Discussion

The purpose of this study was to examine the psychometric properties of the Competition Scale of the TOPS in Greek athletic population. The TOPS-CS is designed to assess the psychological strategies used by athletes in competition, thus giving valuable information to coaches and practitioners about the psychological parameters underlying athletic performance.

In the present study, results differentiate a lot depending on the athletes’ age group. In the first study, for athletes aged 16–20 years, exploratory factor analysis produced an acceptable eight factor structure, a result also found in other studies (Jackson et al., 2000; Taylor et al., 2000). The eight factors hypothesized to underlie the items were: self-talk, emotional control, automaticity, goal-setting, imagery, relaxation, activation and negative thinking. In the exploratory factor analysis, all factors were obtained. It is noteworthy to mention that at the individual item level two items showed very good to excellent loadings on their hypothesized factor. Two items showed good loadings, two fair loadings and there was no item with a weak loading. Weak factor loadings may indicate that participants did not understand the exact meaning of an item or that the item is not reflecting exactly the concept of the factor that it is meant to represent (Comrey & Lee, 1992). Results from reliability analysis also provided adequate evidence for the internal consistency and stability of the scale. Furthermore, confirmatory factor analysis supported the initial structure of the inventory for the overall model. More specifically, emotional control, goal-setting, imagery, negative thinking and relaxation subscales showed good fit whereas activation, self-talk, and automaticity, less so. Overall, it appears that TOPS-CS can be used in Greek athletic population older than 15 years of age.

However, in the second study, the use of TOPS in Greek athletic population aged 12 to 15 years did not demonstrate adequate psychometric properties. Exploratory factor analysis did not support the initial eight-factor structure of the inventory for Greek athletes of this age range. Seven factors were produced with inadequate content validity and poor factor loadings. In particular, items from the subscale of goal setting are loading in other factors. For example, in the factor of goal-setting, the item “I evaluate whether I achieve competition goals” is loading on the factor of activation. A possible explanation of this result could be that the ability of setting goals in competition is developing after some years of athletic experience and athletes of this age are not able of at least expressing it. In addition, young athletes in some sports probably cannot set goals and pursue them independently from their parents and coaches’ influence. Besides, young athletes are not familiar with the concepts of the TOPS items and they have not enough experience and knowledge of the use of psychological skills. McCarthy et al. (2010) in their investigation with young adolescent athletes (10–15 years old), found that young athletes were less able to explain the meaning of self-talk and relaxation, while they could better understand goal-setting and imagery.

According to Lane et al. (2004), the language used in some items is possibly inappropriate for young athletes in order to understand clearly the exact meaning of the items. For example, in the factor of automaticity the item “Don’t think about performing much-just let it happen” might be interpreted by young athletes more like indifference and/or unconcern and less like ability to compete with the least mental processing of the movements. Despite the good factor loadings of the automaticity subscale, it was not always clear to participants what the items meant. Indeed, the automaticity factor in the competition subscale still requires further attention, as also suggested by Thomas et al. (1999). It seems that young athletes need suitable training in order to understand psychological skills and incorporate these skills in practice and competition (Stallard, 2005).

Another possible explanation of the results of the present study could be that the age group 12–15 years was too broad (158 athletes were 12–13 and 175 athletes were 14–15 years old) and included subgroups of different stages of development. During this critical period of development, young athletes experience rapid physiological, neurologic, and psychological growth, which does not follow the same pattern for all of them. According to Malina and Bouchard (1991), even children of the same chronological age may differ by several years in their biological maturation.

Further research should examine the psychometric properties of the TOPS with a modified study design, dividing the athletes’ group of 12–15 years into subgroups, in order to examine the performance strategies used by athletes of different developmental stages and to explore their psychological skills in relation to their competing experience.

In conclusion, the results of the first study provide adequate evidence of the psychometric properties of the Test Of Performance Strategies-Competition Scale for Greek adult athletic population. TOPS-CS is a valid and reliable instrument for use in athletes aged 16 to 20 years. However, for athletes aged 12–15 years, question marks remain over some aspects of the factorial validity of the instrument and its appropriateness for young athletes remains in doubt. In addition, in future studies it would be useful to examine the psychological skills and strategies used by athletes in different sports (individual or team sports, open-closed activities) and levels of athletic development.

## Figures and Tables

**Table 1:** Exploratory factor analysis of the Test of Performance Strategies-Competition Scale (group 1=382 athletes)

Items		Factors								Communalities
		1	2	3	4	5	6	7	8	
22		.81								.70
23		.80								.72
21		.69								.60
24		.67								.50
	18		.90							.84
	19		.87							.80
	17		.83							.76
	20		.57							.40
2				.83						.78
1				.82						.77
3				.76						.62
4				.66						.51
	27				.83					.77
	25				.81					.72
	26				.80					.70
	28				.56					.55
29						.82				.69
31						.75				.70
30						.69				.51
32		.32				.68				.68
	7						.77			.65
	5						.73			.60
	6					.31	.67			.62
	8						.65			.52
14								.77		.69
13								.73		.63
15								.68		.55
16		.36						.45		.44
	12								.80	.65
	11								.68	.52
	9								.66	.55
	10							−.42	.51	.48
Eigenvalues		9.03	8.89	8.53	8.19	7.90	7.51	7.10	6.13	
%Variance		9.03	17.91	26.45	34.64	42.54	50.05	57.14	63.27	

**Table 2:** Internal consistency and reliability of the TOPS-CS

Subscales	Inter items correlations (group 1=382)	Cronbach *α* (group 1=382)	Test-Retest (n=120)
Self-talk	.34	.81	.77
Emotional control	.13	.75	.80
Automaticity	.18	.63	.67
Goal setting	.41	.72	.73
Imagery	.26	.84	.69
Activation	.37	.80	.64
Negative thinking	.37	.82	.81
Relaxation	.16	.78	.78

**Table 3:** Confirmatory factor analysis of the subscales of the TOPS-CS (group 3=288 athletes)

**Subscales**	**Χ^2^** **(df=2)**	**TLI**	**CFI**	**RMSEA**
Self-talk	9.9 p=0.007	0.966	0.989	0.104
Emotional control	1.2 p=0.538	1.012	1.000	0.000
Automaticity	12.3 p=0.002	0.674	0.935	0.119
Goal setting	5.6 p=0.061	0.941	0.988	0.070
Imagery	3.0 p=0.0227	0.996	0.999	0.036
Activation	17.4 p=0.000	0.908	0.969	0.145
Negative thinking	7.0 p=0.030	0.964	0.988	0.083
Relaxation	1.8 p=0.409	1.002	1.000	0.000
